# Overweight or Obese Individuals at Eighteen Years of Age Develop Pancreatic Adenocarcinoma at a Significantly Earlier Age

**DOI:** 10.1155/2018/2380596

**Published:** 2018-06-05

**Authors:** David T. Chao, Nilesh H. Shah, Herbert J. Zeh, Aatur D. Singhi, Nathan Bahary, Kevin M. McGrath, Kenneth E. Fasanella, Amer H. Zureikat, David C. Whitcomb, Randall E. Brand

**Affiliations:** ^1^Department of Medicine, University of Pittsburgh Medical Center, Pittsburgh, PA, USA; ^2^Division of Gastroenterology and Hepatology, Kaiser Permanente, Los Angeles Medical Center, Los Angeles, CA, USA; ^3^Department of Dental Public Health, University of Pittsburgh, Pittsburgh, PA, USA; ^4^Division of Gastrointestinal Surgical Oncology, University of Pittsburgh Medical Center, Pittsburgh, PA, USA; ^5^Department of Pathology, University of Pittsburgh Medical Center, Pittsburgh, PA, USA; ^6^Division of Hematology/Oncology, University of Pittsburgh Medical Center, Pittsburgh, PA, USA

## Abstract

**Background:**

Adolescent obesity is a national epidemic that recently has been shown to increase risk for pancreatic adenocarcinoma (PC) and is associated with an earlier age of PC onset. We hypothesized that PC patients who are overweight or obese at age 18 would have an earlier age of PC onset.

**Methods:**

Retrospective review of 531 patients in our PC registry was completed. Self-reported weight at age 18 and maximum lifetime weight were used to calculate body mass index (BMI) at age 18 (BMI-18) and maximum lifetime BMI.

**Results:**

Complete BMI and baseline covariate data was available in 319 PC patients. Mean age (in years) of PC diagnosis for patients whose BMI-18 was overweight (64.0) or obese (59.9) was significantly different when compared to patients with a normal BMI-18 (66.7). No significant difference was observed in the mean age of PC diagnosis in those patients who maintained a normal BMI-18 when compared to those patients who subsequently became overweight or obese (67.0 versus 66.6; *p* = 0.65).

**Conclusions:**

An elevated BMI at age 18 is associated with an earlier age of PC onset and should be factored into determining the optimal age of beginning screening for patients at high risk for PC.

## 1. Introduction

Pancreatic adenocarcinoma (PC) is currently the third leading cause of cancer death among men and women in the United States with 44,330 expected deaths in 2018 [1]. Of the top five causes of cancer mortality—lung, colorectal, pancreas, breast, and prostate—only the number of deaths attributed to PC is projected to steadily rise to become the second leading cause of cancer death by 2030 [2]. The 5-year survival rate of PC is a dismal 8% and reflects the typically advanced stage of disease at time of diagnosis [1]. Despite a median survival time of 6 months, recent clonal studies indicate PC develops slowly over many years [3] and suggest there is an opportunity to potentially improve prognosis through detection of subclinical malignant pancreatic lesions prior to the development of metastatic disease.

While the relatively low incidence of PC makes it impractical to screen the general United States population, experts agree surveillance of high-risk individuals is warranted [4, 5]. Presently, the only candidates considered for surveillance are individuals with an underlying genetic condition recognized to increase risk for developing PC and members of families with a hereditary predisposition for developing PC [5]. However, there is no expert consensus regarding when to ideally begin surveillance in these high-risk individuals or how to factor modifiable PC risk factors such as smoking, heavy alcohol consumption, and obesity into surveillance strategies.

Obesity is associated with an increased risk for at least 13 types of cancer [6], including colorectal [7], breast [8], endometrial [9], kidney [10], thyroid [11], esophageal [12], liver [13], and pancreatic [14]. Additionally, growing evidence has also specifically associated adolescent and early adulthood obesity with an increased cancer risk [13, 15]. In 2009, a case-control study involving 841 patients with PC demonstrated individuals who were overweight between the ages of 14 and 39 years or obese between the ages of 20 and 49 years were at increased risk of developing PC [16]. Furthermore, individuals who were overweight or obese between the ages of 20 and 49 years had an earlier onset of PC by 2 to 6 years, respectively, compared with PC patients of normal weight during this same time period [16]. These observations are particularly concerning given that the number of obese infants and young children globally is expected to increase from 42 million in 2013 to 70 million in 2025 at current trends [17]. In the United States, data from the National Health and Nutrition Examination Survey indicates 70.7% of adults aged 20 years and older and 33.4% of youth between ages 2 and 19 years were overweight or obese in 2013-2014 [18].

Based on the above, we hypothesized that patients who were overweight or obese at age 18 in our institution's PC registry would have an accelerated course of pancreatic carcinogenesis resulting in a younger mean age of PC diagnosis. In addition, we aimed to evaluate how weight gain following age 18 affected the age of PC diagnosis.

## 2. Materials and Methods

### 2.1. Study Design and Patient Population

We performed a retrospective review of consecutive patients with histologically confirmed pancreatic adenocarcinoma between June 1, 2003, and May 31, 2012, who provided informed consent for enrollment into our Pancreatic Adenocarcinoma Gene Environment Risk (PAGER) study. Patients included in the study were seen at the University of Pittsburgh Medical Center (UPMC) and prospectively recruited into the study database at the time of their PC diagnosis. Although UPMC is a tertiary-care referral center, the vast majority of the patients in our PC registry are locoregional and live within 200 miles of our center. Upon enrollment, patients completed a self-reported medical questionnaire designed for the PAGER study to collect information including the patient's demographic, lifestyle, family, and medical histories.

### 2.2. Data Abstraction and Collection

For each patient considered for inclusion into the present study, we abstracted information including their height and weight at different time points (e.g., at age 18, maximum lifetime weight [excluding pregnancy], maximum weight 2 years prior to PC diagnosis, and at time of enrollment). We also collected information about known PC risk factors that served as covariates for our study: smoking history (active smoker or quit <10 years ago versus never smoker or quit >10 years ago), alcohol consumption (≥3 drinks/day versus <3 drinks/day), diabetes mellitus status (yes versus no), and family history of PC in first-degree relatives (present versus absent). Manual chart review was performed to confirm the patient's self-reported information. Those with incomplete covariate data or covariate histories that could not be confirmed during chart review were excluded from the study.

Standard categorizations of body mass index (BMI) as defined by the World Health Organization were used to allocate patients into three primary cohorts of normal (BMI: 18.5–24.9 kg/m^2^), overweight (BMI: 25.0–29.9 kg/m^2^), and obese (BMI: ≥30.0 kg/m^2^) according to their self-reported height and weight at age 18 (e.g., BMI-18). We excluded patients whose BMI-18 was less than 18.5 kg/m^2^.

The patient's reported maximum weight was used to calculate their maximum lifetime BMI (e.g., BMI-Max). For patients with a normal BMI at age 18, we determined the subsequent development of obesity as an adult by stratifying these patients into normal, overweight, and obese cohorts defined by their BMI-Max.

Surgical pancreatectomy specimens were subject to gross examination and sectioning as per a standardized protocol established at the UPMC Department of Pathology. For each specimen, the entire neoplasm and all the peripancreatic soft tissue to include regional lymph nodes were submitted for tissue processing. In addition, representative sections of the patient's background pancreas, stomach, duodenum, and spleen, depending on surgical specimen, were taken for histopathologic examination. Each tissue section was processed through formalin fixation and paraffin embedding and, finally, evaluated by a trained pancreatobiliary pathologist as part of routine clinical care.

### 2.3. Statistical Analysis

We first looked for differences in the age of PC diagnosis based on BMI status. We used the group that had normal BMI at age 18 as baseline and used multiple linear regression to evaluate associations between mean age of PC diagnosis and BMI, comparing the normal group to the overweight at age 18 group, the obese at age 18 group, and the combined overweight/obese group. The analyses were adjusted for smoking, alcohol, diabetes status, family history of PC, and gender. We then performed a subset analysis on the subjects with a normal BMI-18. Using the group whose maximum BMI remained normal as baseline, we used multiple linear regression to compare the age of PC diagnosis to the groups that became overweight and obese later in life. We again adjusted for smoking, alcohol, diabetes status, family history of PC, and gender. We then used multivariable regression to evaluate the effects of smoking history and age of maximum BMI on age of PC diagnosis for subjects who were normal BMI at age 18 then became overweight or obese later in life. We also use multivariable regression to evaluate the effects of smoking history and age of maximum BMI on age of PC diagnosis for subjects with a normal BMI at age 18 and remained normal until their time of PC diagnosis. Statistical analyses were performed using Stata software version 14 (StataCorp LP, College Station, TX), with statistical significance set at *p* < 0.05.

## 3. Results

### 3.1. Patient Demographics

**Figure 1:**
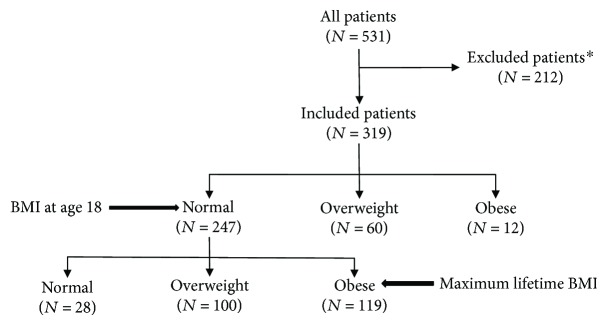
Patient distribution. ^∗^Patients excluded due to incomplete covariate data or BMI at age 18 <18.5 kg/m^2^.

We identified 531 patients with histologically confirmed pancreatic adenocarcinoma during a 9-year period beginning in June 2003. Incomplete covariate data led to the exclusion of 211 patients while a BMI-18 of <18.5 kg/m^2^ led to the exclusion of one additional patient. No significant difference was observed between patients who were excluded and patients included in analysis in the following variables: gender, family history, age of diagnosis, DM status, or alcohol. The rate of smoking was higher in the group that participated, but the group with missing data had a higher rate of missing smoking information. The remaining 319 patients included for analysis did not demonstrate any significant difference with respect to baseline covariates (Table 1) and were divided into primary BMI-18 cohorts and secondary BMI-Max cohorts as depicted in Figure 1.

### 3.2. BMI-18 and Age of PC Diagnosis

Patients were categorized into normal, overweight, or obese cohorts according to their BMI at age 18. As shown in Table 2, the mean ages of PC diagnosis for patients with an overweight BMI-18 (64.0 years old) and obese BMI-18 (59.9 years old) were both significantly earlier than the mean age of PC diagnosis for individuals who had a normal BMI-18 (66.7 years old).

### 3.3. Age of PC Diagnosis and Weight Gain among PC Patients with a Normal BMI-18

Among 247 patients with a normal BMI-18, 100 subsequently became overweight and 119 developed obesity. No significant difference was observed in the mean age of PC diagnosis in those who became overweight or developed obesity following age 18 when compared with those whose BMI remained normal (Table 3). Piecewise regression demonstrated no significant change in the mean age of PC diagnosis among patients whose BMI-Max increased up to 10 units from their BMI-18.

We analyzed mean age of PC diagnosis based on absolute BMI increase between BMI-18 and BMI-Max for all patients included for analysis. No significant difference was observed in the mean age of PC diagnosis between patients who had less than a 3-unit BMI increase (67.4 years old) and those who had greater than a 7-unit BMI increase (66.6 years old) (Table 4).

### 3.4. Smoking and Obesity Status on Mean Age of PC Diagnosis

We examined the effect of smoking history for different categories of obesity.  Table 5 shows the age of PC diagnosis for the groups that were overweight or obese at 18, normal at 18 then became overweight/obese, and remained normal, after adjusting for baseline covariates. The adjusted analysis showed that for the normal always group, a positive smoking history was associated with a 7.7-year earlier PC diagnosis (*p* = 0.16). For the group with a normal BMI-18 then became overweight/obese, a positive smoking history was associated with a 4.6-year earlier PC diagnosis (*p* = 0.014). For the group that was overweight/obese at 18, a positive smoking history was associated with a 4.2-year earlier PC diagnosis (*p* = 0.15). The regression model also showed that having a positive smoking history leads to, on average, an approximately 5-year earlier PC diagnosis when controlling for all other factors.

### 3.5. Histologic Evaluation

Three patients with an obese BMI-18 did not receive preoperative chemotherapy and had tissue specimens available for reexamination. These patients were matched for smoking history, alcohol use, diabetes status, family history of PC, and absence of preoperative chemotherapy exposure. Compared with patients who had a normal BMI-18, PC patients who were obese at age 18 were observed to have slightly more PanIN-2 and PanIN-3 lesions present, but the sample size was too small to perform meaningful statistical analyses.

## 4. Discussion

The results from our study find an increased BMI-18 (≥25 kg/m^2^) significantly decreases the mean age of PC diagnosis and suggest that an earlier onset of excess adiposity is a critical factor for developing PC at a younger age. We found PC patients who are overweight or obese at age 18 are diagnosed approximately 3 and 7 years younger, respectively, compared to PC patients with a normal BMI-18. Nonoverweight individuals at age 18 who go on to become overweight were not diagnosed with PC at a significantly different age compared to PC patients who never become overweight.

In 2007, the World Cancer Research Fund Panel reported there was “convincing” increased risk of PC related to “body fatness” and a “probable” increased risk with “abdominal fatness” [19]. Since then, mounting evidence has further affirmed the association between obesity and PC, including the National Institutes of Health-AARP Diet and Health Study, which concluded excess adiposity at any adult age was associated with an increased risk of PC and that morbidly obese adults (BMI ≥ 35) had a 45% greater PC risk compared with those with a normal BMI [20, 21]. Obesity may not only increase risk for developing PC but also contribute to a poorer prognosis and increased all-cause mortality [16, 21, 22]. More recently, there has been growing evidence associating adolescent and early adulthood obesity with increased risk of gastrointestinal malignancies including esophageal [23], gastric [24], colorectal [15, 25], liver [13], and pancreatic [16, 26, 27]. Our conclusion that overweight and obese individuals at age 18 are diagnosed with PC 3 and 7 years younger is similar to the findings reported by Li et al., in which overweight or obese individuals between the ages of 20 and 49 years were diagnosed with PC approximately 2 and 6 years earlier [16]. A pooled analysis of 14 cohort studies involving 2135 individuals with PC also concluded that excess weight between ages 18 and 21 resulted in a slightly younger mean age at PC diagnosis [28]. These and other studies were considered in the 2012 “Continuous Update Project” report in which the World Cancer Research Fund Panel agreed “greater childhood growth”—which considers BMI at around 20 years old among other factors—and “body fatness” are “probable” and “convincing” causes of PC [29].

While the mechanistic relationship between obesity and pancreatic carcinogenesis is likely multifactorial, activation of oncogenic Kras is observed in more than 90% of precursor pancreatic intraepithelial neoplastic lesions and is widely accepted as the initial step in the malignant transformation of normal pancreatic acinar cells [30]. Activation of oncogenic Kras leads to perpetuation of downstream signaling pathways, including proinflammatory pathways that may further activate Kras in a reinforcing, positive feed-forward loop [31]. Both high-fat diets and excess adiposity may provide an inflammatory environmental stimulus to trigger this pathway [32] and could explain the progressively younger age of PC diagnosis observed among PC patients in the current study who were overweight or obese at age 18. Whether persistent Kras activity is due to constitutive activation [33] or repetitive local stimuli [34] remains in question, but once oncogenic Kras is triggered, there appears to be a steady progression toward inflammation, fibrosis, and tumor development.

Our study emphasizes the importance of maintaining a healthy body weight and avoiding excess weight gain during adolescence. Obesity that begins during childhood is likely to persist into adulthood and increases risk for developing associated comorbid diseases, including cancer [35]. Whether intentional weight loss following obesity onset may delay the course of carcinogenesis remains unknown. Weight reduction may reduce cancer risk through decreased circulating levels of proinflammatory cytokines and other cancer-associated protein biomarkers [36]. In the present study, however, among 72 patients who were overweight or obese at age 18, no significant difference was observed in the mean age of PC diagnosis between the 7 patients whose BMI decreased by 5 units after age 18 and the 65 patients who did not experience such a significant reduction in weight prior to diagnosis with PC. Furthermore, aside from studies suggesting that weight loss in postmenopausal women may be protective against breast cancer [37], to our knowledge, there is otherwise no evidence suggesting intentional weight loss during adulthood may delay the development of cancer. If weight reduction following obesity onset does not significantly alter an individual's risk and course of carcinogenesis, then greater emphasis must be placed on maintaining a healthy body weight during childhood.

Although our sample size was limited, histological review of pancreatic tissue samples from 3 patients with an obese BMI-18 revealed a trend towards an increase in the number of observed precancerous PanIN-2 and PanIN-3 lesions compared to matched controls with a normal BMI-18. To our knowledge, this is the first study providing histologic evidence that advanced PanIN lesions are observed more often among those who were overweight or obese during childhood and suggests pancreatic tumorigenesis may occur earlier in these individuals. Our finding is consistent with a recent study demonstrating obesity increases risk for development of precancerous PanIN lesions [38]. Further large-scale studies will be needed to validate this finding.

Experts agree surveillance of high-risk individuals is beneficial, although who should be screened for PC and when to initiate surveillance remains an ongoing discussion [5]. Population-based screening is not recommended due to the relatively low estimated lifetime risk of developing PC in average-risk individuals. In contrast, surveillance should be considered for an enriched cohort of high-risk individuals with a known increased lifetime risk of developing PC as compared to the general population [5]. The significantly increased risk among those with a hereditary predisposition led members of the International Cancer of the Pancreas Screening (CAPS) Consortium to reach a consensus agreement that first-degree relatives of an affected PC patient from a familial pancreatic cancer kindred, patients with Peutz-Jeghers syndrome and p16, BRCA2, and HNPCC mutation carriers with at least one affected first-degree relative should undergo PC surveillance [5].

In contrast to genetic risk factors, modifiable PC risk factors such as smoking [39], alcohol use [40], obesity [14], and diabetes mellitus [41] increase an individual's relative risk of developing PC approximately 1 to 3-fold. Factors including smoking and alcohol use exhibit a variable effect often related to the magnitude of exposure and may substantially decrease the age of PC onset either alone [42] or in conjunction with other known PC risk factors [43]. For example, smoking has been demonstrated to lower the age of PC diagnosis approximately 15–20 years among patients with hereditary pancreatitis [44]. Likewise, smoking and alcohol have been associated with early onset PC in a dose-dependent manner [42, 45]. In the current study, smoking had a statistically significant impact in lowering the mean age of PC diagnosis among patients who had a normal BMI-18 and became overweight or obese. In patients whose BMI categorization did not change between BMI-18 and BMI-Max, smoking lowered the mean age of PC diagnosis between 4.2 and 7.7 years in patients. Although the age difference in these two groups did not achieve statistical significance, we believe this was a consequence of the small sample size. These results suggest that smoking also significantly lowers the mean age of PC diagnosis in a manner that is independent of a patients' adolescent and adult BMI status.

Apart from smoking and adolescent obesity, no other PC risk factor appeared to significantly lower the mean age of PC diagnosis in the present study. Obesity appears to trigger a progressive course of carcinogenesis, leading to an earlier age of PC diagnosis when excess weight gain occurs during adolescence. While obesity itself is an established risk factor for developing PC, the results of our study suggest adult-onset obesity does not appear to significantly lower the age of PC diagnosis in the same manner as childhood obesity. That adolescent obesity not only raises an individual's risk for developing PC but also leads an earlier age of PC diagnosis carries alarming public health implications and provides further urgency to finding effective solutions to reduce childhood obesity both nationally and internationally.

Our study provides supporting evidence that an earlier age of obesity leads to early onset PC and that BMI at age 18 should be considered as a potential factor in identifying age for initiating PC surveillance in appropriate candidates. However, our study is not without limitations. UPMC is a tertiary-care center and therefore subject to referral bias with a greater percentage of patients presenting with early-stage cancer. However, the vast majority of our pancreatic cancer patient population is locoregional with more than 90% living within 200 miles of our center. Our use of self-reported weights is subject to recall bias and an inherent degree of inaccuracy, even if prior studies have validated the accuracy of self-reported weights [46]. Our study had a small number patients in our obese BMI-18 cohort, which is reflective of and consistent with the significantly lower proportion of obese individuals in the general United States population approximately 40–45 years ago [47]. Our analysis is also limited by not knowing the age when patients became overweight or obese. We also acknowledge nearly 40% of the patients in our PC registry were excluded from the study analysis in large part due to the lack of available complete covariate data. We examined the cohort of patients excluded from analysis and did not find any statistically significant differences between the patients who were excluded and those who were included for analysis. Finally, we acknowledge that we may not have controlled for other potentially confounding PC risk factors including waist-to-hip ratio, waist circumference, physical activity, and dietary habits.

## 5. Conclusions

In summary, individuals who develop PC are likely to be diagnosed at a significantly younger age if they were overweight or obese at age 18. BMI at age 18 may be useful for determining when to initiate surveillance in high-risk individuals. Further studies are needed to confirm the clinical and histopathological evidence and discern the optimal age to query for assessing the risk of early onset obesity to developing PC.

## Figures and Tables

**Table 1:** Baseline patient demographics.

Variable	Overall (*N* = 319)	BMI-18 classification			*p* value
		Normal (*N* = 247)	Overweight (*N* = 60)	Obese (*N* = 12)	
Age of pancreatic cancer diagnosis (y, mean ± SD)	65.9 ± 9.9	66.7 ± 10.0	64.0 ± 9.2	59.9 ± 8.6	0.02
Sex [*N* (%)]					0.27
Male	178 (55.8)	133 (53.9)	39 (65.0)	6 (50.0)	
Female	141 (44.2)	114 (46.1)	21 (35.0)	6 (50.0)	
Smoking history [*N* (%)]					0.63
Yes	264 (82.8)	204 (82.6)	51 (85.0)	9 (75.0)	
No	55 (17.2)	43 (17.4)	9 (15.0)	3 (25.0)	
Alcohol status [*N* (%)]					0.29
Heavy (>3 drinks/day)	68 (21.7)	50 (20.6)	17 (28.3)	1 (9.1)	
Light (≤3 drinks/day)	246 (78.3)	193 (79.4)	43 (71.7)	10 (90.9)	
Diabetes status [*N* (%)]					0.51
Yes	131 (41.2)	97 (39.4)	28 (46.7)	6 (50.0)	
No	187 (58.8)	149 (60.6)	32 (53.3)	6 (50.0)	
Family history of PC [*N* (%)]	29 (9.2)	21 (8.6)	6 (10.2)	2 (16.7)	0.44

**Table 2:** Mean age of pancreatic cancer diagnosis according to BMI at age 18.

	N	Mean age (SD)	Mean difference^1^	*p* value^2^
BMI at age 18	319			
Normal	247	66.7 (10.0)		
Overweight/obese	72	63.3 (9.2)	−3.75 (−6.35, −1.16)	0.01
Overweight	60	64.0 (9.2)	−3.10 (−5.89, −0.30)	0.03
Obese	12	59.9 (8.6)	−7.26 (−13.26, −1.25)	0.02

^1^Estimated by linear regression with PC diagnosis as a dependent variable and BMI group as an independent variable, adjusting for smoking history, alcohol consumption, gender, family history of PC, and diabetes status. 95% confidence intervals are presented. ^2^Comparison of the BMI group with the normal group as baseline.

**Table 3:** Mean age of pancreatic cancer diagnosis according to BMI in patients with a normal BMI at age 18.

	*N*	Mean age (SD)	Mean difference^1^	*p* value^2^
Normal BMI at age 18	247			
Remain normal	28	67.0 (10.5)		
Become overweight/obese	219	66.6 (10.0)	−0.96 (−5.05, 3.13)	0.65
Overweight	100	67.5 (10.6)	−0.04 (−4.65,4.58)	0.99
Obese	119	65.9 (9.5)	−1.01 (−5.27, 3.25)	0.64

^1^Estimated by linear regression with age of PC diagnosis as a dependent variable and BMI group as an independent variable, adjusting for smoking history, alcohol consumption, gender, family history of PC, and diabetes status. 95% confidence intervals are presented. ^2^Comparison of BMI group with normal group as baseline.

**Table 4:** Mean age of PC diagnosis according to absolute BMI increase between BMI at age 18 and maximum lifetime BMI.

BMI increase	Mean age (years)	*p* value
<3 units	67.4	0.967
3–5 units	65.9	
5–7 units	67.0	
>7 units	66.6	

**Table 5:** Mean age of PC diagnosis based on BMI status and smoking history.

	Overweight/obese at 18	Normal at 18, become overweight/obese	Normal at 18, remain normal
No/remote history of smoking^1^	67.3 (*n* = 12)	70.1 (*n* = 39)	77.0 (*n* = 4)
Active/recent smoking history^2^	62.5 (*n* = 60)	65.9 (*n* = 180)	65.4 (*n* = 24)
*p* value	0.15	0.014	0.16

1 includes individuals who have never smoked or those who have quit smoking more than 10 years prior to their pancreatic cancer diagnosis. 2 includes individuals who were still smoking at the time of their pancreatic cancer diagnosis as well as those who quit smoking less than 10 years prior to their pancreatic cancer diagnosis

## Data Availability

The data used to support the findings of this study are housed at the University of Pittsburgh or are available from the corresponding author upon request.
